# Laparoscopic Sacropexy: A Retrospective Analysis of the Subjective Outcome in 310 Cases

**DOI:** 10.1155/2012/538426

**Published:** 2011-09-07

**Authors:** B. Bojahr, G. Tchartchian, M. Waldschmidt, R. Ohlinger, R. L. De Wilde

**Affiliations:** ^1^Klinik für Minimal Invasive Chirurgie, Kurstraße 11, 14129 Berlin, Germany; ^2^Medizinische Fakultät, Ernst-Moritz-Arndt-Universität Greifswald, Friedrich-Loeffler-Straße 23 b, 17457 Greifswald, Germany; ^3^Klinik für Frauenheilkunde, Pius-Hospital, Georgstraße 12, 26121 Oldenburg, Germany

## Abstract

*Background and Objective*. The aim of this study was to assess the subjective outcome following laparoscopic sacropexy. *Methods*. We performed a retrospective cohort study among women treated for descensus with laparoscopic sacropexy between January 2000 and December 2007. 310 patients received questionnaires during followup assessing major pre- and postoperative symptoms and overall satisfaction. *Results*. 214 (69%) patients responded to the questionnaire. Mean followup was 24.5 months. The number of patients with back or lower abdominal pain, foreign body sensation in the vagina and prolapse-related symptoms, urinary symptoms, vaginal and bladder infections, and the need for pessary usage decreased significantly postsurgically. Bowel symptoms increased slightly but not significantly. Two years after surgery, nearly 2 thirds of the women were satisfied or very satisfied with the outcome. *Conclusion*. Laparoscopic sacropexy is an effective treatment of descensus, with favorable or satisfactory subjective outcomes.

## 1. Introduction

Pelvic organ prolapse is highly prevalent in women (30–50%) [1], with a lifetime risk of surgical repair of 11% [2]. It is associated with high costs for its treatment and a significant drop of women's quality of life and serious discomfort [3]. The cause of prolapse is not known, but risk factors include age, increasing number of gravidity and (especially) vaginal deliveries, obesity, chronic cough, constipation, and a history of hysterectomy [1, 4]. Associated symptoms are amongst urinary and fecal incontinence, vaginal ulceration, problems with defecation, and sexual dysfunction [1]. 

Often pelvic symptoms and the extent of prolapse are weakly correlated [5, 6]. Women who are most impaired seek surgical solutions. The main intention of surgery is to relieve or improve prolapse symptoms and, if possible, associated urinary and bowel symptoms. There are numerous surgical procedures available for genital prolapse. The success rate of laparoscopic sacropexy has been reported by a number of authors to be 90–98% [7–10], but only a few studies have evaluated the success of prolapse surgery by means of patients' reported symptoms and satisfaction. The aim of this study was to analyze the subjective outcome of laparoscopic sacropexy.

## 2. Material and Methods

A retrospective study was performed including all patients undergoing laparoscopic sacropexy as treatment for descensus between January 2000 and December 2007 at the “Klinik für Minimal Invasive Chirurgie” (Berlin). In this period, 310 patients underwent 310 primary sacropexies and 13 recurrent interventions. Data were received from the medical files of the patients and through evaluation of a patient questionnaire. 

Preoperative data included age, body mass index (BMI), menopausal status, parity, the classification of the patient in accordance with the American Society of Anesthesiologists (ASA) score (I-IV), the classification of pelvic organ prolapse in accordance with the DGGG guidelines issued by the German Association for Gynecology and Obstetrics [11], descensus of the anterior and posterior compartment, history of previous gynecological operations, and respective leading symptoms. It was also documented to what extent additional surgical interventions became necessary during laparoscopic sacropexy. 

The indication for laparoscopic surgery as well as for simultaneous surgery, depending on concomitant diseases, was set by the respective surgeon in the pre-operative consultation. Simultaneous operations were not considered as exclusion criteria.

### 2.1. Presurgical Preparations

No laxative preparation was done. On induction of anesthesia, the urinary bladder was catheterized, and, after vaginal disinfection, dressing forceps with a fixed swab were inserted into the vagina.

 Ten patients (3.2%) were permanently catheterized due to additional vaginal treatments. A total of 46 patients (14.2%) prophylactically received one dose of 1.5 g ampicillin/sulbactam intraoperative and postoperative.

### 2.2. Surgical Technique

In laparoscopic sacropexy according to Raatz, as it is performed at the Klinik für Minimal Invasive Chirurgie (Clinic for Minimal Invasive Surgery) in Berlin since 2000, the repositioning of the prolapsed anatomical structures is accomplished by employing a prolene mesh. The mesh is attached to either the prolapsed vaginal stump or the posterior cervical wall, in case of uterus prolapse and its planned preservation or to the cervical stump—following supracervical hysterectomy. After the repositioning of the prolapsed structures, the mesh is attached to the promontorium.

After bladder catheterism and vaginal disinfection, dressing forceps with a fixed swab are inserted into the vagina at the beginning of the operation. With the patient in horizontal position, with stretched legs, a CO_2_ pneumoperitoneum is established utilizing a Veress needle. A 5-mm transumbilical trocar is used for the laparoscopy with 5 mm/30° optics. Then the patient is placed in maximum (steep) Trendelenburg's position. The laparoscopic sacropexy then requires only two more 5 mm puncture sites in the lower abdomen. The left *incision* is made lateral to the epigastric vessels above the pubic hair line on the left side, while the right trocar is inserted considerably higher, that is, lateral to the promontorium or even slightly higher.

In order to improve the exposure of the promontorium, it may be helpful to position the patient slightly to the left, or to remove the sigma from the operating area through a restraining suture on the mesosigma. In case of an existing right adnexa, this may also be temporarily removed from the operating area by using a PDS sling. After exposure and palpation of the promontorium using grasping forceps, bleeding is controlled by bipolar coagulation, and the peritoneum is opened up using scissors. On the right side, the ureter and the lateral iliac vessels should be exposed in order to prevent injuries.

The peritoneum is opened up along the right pelvic wall while special caution must be observed regarding the ureter and the vessels of the mesosigma up to the cervix or the vaginal stump, respectively. The The right sacrouterine ligament serves as an orientation mark; that is, the peritoneum is opened up at the upper edge of the ligamentum sacrouterinum up to the cervix, after which the peritoneum is partially dissected from the posterior cervical wall.

After the peritoneum is opened up above the promontorium, the presacral fat tissue is loosened and removed from the Os sacrum using atraumatic grasping forceps on the left and bipolar grasping forceps on the right side. This mostly takes place rather bluntly and must be done very carefully, in order to not rupture any vessels. This way, the flat surface of the ligamentum longitudinale becomes visible bit by bit. 

At this point, it is of utmost importance to avoid a lesion of the median sacral artery. An about 8–10 cm long, and 1.5–2-cm-wide strip of the prolene mesh (SURGIPRO Mesh) is then inserted into the abdomen. To this end, the atraumatic grasping forceps are moved in an outward direction above the 5 mm trocar, after the valve of the trocar has been removed earlier. 

The mesh is then grasped outside the abdominal cavity and is pulled into the abdominal cavity via the 5 mm trocar while still folded together. This is followed by the fixation of the mesh on the cervical stump. The mesh is attached on broadbase to the cervix through a continuous suture. This suture requires several stiches into and out of the cervix and the mesh. The mesh is then fixated by tying several knots. Alternatively, fixation is also possible through simple interrupted stiches. We mostly use a POLYSORB 1. Then follows the insertion of a second thread and needle (ETHIBOND 0). The ligamentum longitudinale is exposed once more. This usually requires holding aside the peritoneum or intestine with the grasping forceps. 

On the right, the needle is clamped in the needle holder, and a Z-suture is stitched onto the ligamentum longitudinale. It must be ensured that the distance between stiches on the ligamentum longitudinale is sufficiently wide so that the mesh does not rip off after fixation. 

The swab placed in the vagina beforehand is then used to move the cervix from a vaginal position to a position above the levator plate. The atraumatic grasping forceps are used to pull the mesh cranially along the Z-suture on the ligamentum longitudinale. The mesh is then stitched several times in line with the promontorium and tied to the ligamentum longitudinale. 

Excess parts of the mesh and thread are cut off and disposed of. Then follows the peritonealization of the cervical stump and the mesh through a continuous suture. This technique of sacropexy may be carried out on the posterior cervical wall in case of uterus preservation, on the vaginal stump in case of previous hysterectomy, or, as described, on the cervical stump during or after a supracervical hysterectomy. Fixation on the vaginal stump takes place after previous preparation at the posterior vaginal wall. However, while pushing up the vaginal stump, with the swab inside the vagina, it must be ensured that the needle does not grasp the swab and stitch it to the surface. In case of uterus preservation, the mesh is fixated on the posterior cervical wall [12].

### 2.3. Questionnaire

Follow-up data were collected through a self-administered questionnaire during postoperative follow-up. All patients received a questionnaire 6 months after surgery at the earliest. The questionnaire used in this study was composed of different international questionnaires, for example, the Pelvic Floor Distress Inventory and Pelvic Floor Impact Questionnaire of Life [13–15]. It included questions on parity (number of children, method of delivery), preoperative menopausal status, following gynecologic surgeries (number and which kind of surgery: laparoscopy, laparotomy, bladder surgery, surgery on the ureter, vaginal surgery on the pelvic floor), and preoperative and postoperative symptoms as lower abdominal pain (staging: mild, medium, severe, very severe), defecation problems (constipation, pain during defecation, fecal incontinence), foreign body sensation in the vagina, dyspareunia, urine incontinence symptoms, voiding difficulties, micturition problems (frequent micturition (more than 3 times a day)), frequent urgency, prolapse-related symptoms, vaginal and bladder infections, back pain, pessary usage. Furthermore, postsurgical changes in frequency of sex, willingness to be examined again by the same surgeon, and overall satisfaction (staging: not satisfied, partly satisfied, satisfied, very satisfied) were evaluated.

### 2.4. Statistical Analysis

All analyses were performed with software packages Exel (Microsoft, Redmond, USA) and the Statistical Package for the Social Sciences (Windows version 15.0; SPSS Inc, Chicago [IL], US). Continuous data were recorded as mean values and standard deviation; the confidence interval was determined using the *t*-test. Statistical significance was demonstrated using the paired-sample Wilcoxon's rank test. The significance level was set at a *P* value of less than 0.05.

## 3. Results

### 3.1. Demographic Data

From January 2000 to December 2007, 310 women suffering from descensus underwent laparoscopic surgery at the “Klinik für Minimal Invasive Chirurgie” (Berlin).  Table 1 shows preoperative demographic and clinical characteristics of the study population.

In the study group, mean parity was 1.85. 64.6% of women were multipara. 92.6% of the children were delivered spontaneously, 2.8% were given birth by forceps delivery or vacuum extraction; respectively, and 1.7% caesarian sections were performed.

Regarding previous gynecologic operations, 43 patients (13.9%) had undergone previous pelvic floor repair surgery because of pelvic organ prolapse. In all cases, vaginal surgeries were performed. 25.5% (79 patients) had previously undergone a hysterectomy (50 vaginal hysterectomies, 18 laparoscopic supracervical hysterectomies (LASH), 11 abdominal hysterectomies). Out of 50 vaginal hysterectomies, 20 surgeries were performed in combination with colporrhaphy, of which were 13 anterior, 6 posterior colporrhaphies, and 1 simultaneous anterior and posterior colporrhaphy. 4 vaginal sacrospinal fixations, 5 surgeries for urine incontinence, including 1 Burch-colposuspension, and 4 tension-free vaginal tape (TVT) operations, were done previously.

### 3.2. Operative Procedures

213 (68.7%), 67 (21.6%), and 30 (9.7%) patients had undergone sacropexy with mesh attachment to the cervix, to the apex of the vagina and to the uterus, respectively.

Concomitant surgeries were necessary for 270 patients (87.1%). Additionally to laparoscopic sacropexy, 195 patients (62.9%) had undergone a LASH, 5 patients (1.6%) had undergone a vaginal hysterectomy, and 1 patient (0.3%) had undergone a laparoscopic vaginal hysterectomy (LAVH). Additionally, 96 adnectomies (31%), 19 salpingectomies (6.1%), 124 bowel and omental adhesiolyses (40%), 5 anterior (1.6%), and 6 posterior (1.9%) colporrhaphies were performed. Twelve women received a TVT, and 30 patients (9.6%) had had ovarian cyst removal surgery.

### 3.3. Residual Operations

Of 323 total sacropexies, thirteen resacropexies (4.0%) were performed. Seven (3.6%) sacropexies with cervical mesh attachment, 4 sacropexies with mesh attachment to the apex vaginae (6%), and 2 sacropexies with mesh attachment to the uterus (6.7%) needed to be operated. In 1 case, a re-resacropexy was necessary after mesh ripping on the distal pole two months after residual operation. Indications for reoperations were descensus level IV in 6 cases (41.7%) and descensus level III in 7 cases (58.3%). Eight surgeries were performed without concomitant intervention; 5 resacropexies were accompanied by simultaneous surgeries.

### 3.4. Questionnaire

Out of 310 patients, 214 patients responded to the questionnaire. Mean time between surgery and answering the questionnaire was 24.5 ± 19.9 months (95% CI 21.6; 27.1, Min/Max 6 months/82 months). Pre- and postoperative results of the questionnaire are presented in Tables 2 and 3.

In sum, 24.7% of the patients subjectively suffered from persistent symptoms 24.5 months after surgery. A significant reduction (*P* < 0.05) after surgery was detected for occurrence of back pain, symptomatic foreign body sensation in the vagina and prolapse-related symptoms, urine incontinence and disturbances of bladder functions like feeling of residual urine and voiding difficulties, the number of vaginal and bladder infections, and the need for pessary usage. Symptoms concerning defecation increased slightly but not significantly after surgery. 

49 patients (out of 202) suffered from preoperative sexual impairment, and 33 patients decided for the operation because of this symptom. While symptoms improved for 39 patients, 6 patients reported an aggravation of symptoms. Of 194 patients, 21.1% reported a change in frequency of sex after surgery, from which 47.1% indicated a reduced and 52.9% an increased frequency of sex. For 78.9% surgery did not influence the frequency of sex. Reduction in frequency of sex correlated with the age of the patients.

After sacropexy, lower abdominal pain and pain severity were reduced significantly (*P* < 0.05). 

In 48 out of 214 patients (22.4%), additional descensus surgeries were necessary during followup. In sum, 66 surgeries, including 9 laparoscopies, 2 laparotomies, 11 bladder surgeries, 7 intestine surgeries, 1 ureter surgery, 14 transvaginal pelvic floor surgeries, and 22 other inventions, were done.

Two years after surgery, nearly two-thirds of the women were satisfied or very satisfied with the outcome, and less than 10% were not satisfied (see Table 4).

No correlation was found between severity of descensus and postoperative satisfaction. No significant difference of satisfaction depending on mesh attachment point was found. Satisfaction correlated with the need for follow-up intervention.

## 4. Discussion

As the correlation between many pelvic symptoms and the extent of prolapse is weak [5, 6], the success of prolapse surgery should not only be assessed by objective outcome but by subjective evaluation through the patient. Subjective success of prolapse surgery is determined by the absence of symptoms. Prolapse causes various and mainly undefined symptoms of urinary, anorectal, and coital nature. In our study, urinary incontinence, defecation problems, dyspareunia, and foreign body sensation in the vagina/prolapse-related symptoms were identified as leading symptoms. By means of these symptoms, subjective success of laparoscopic sacropexy was assessed using a self-administered questionnaire. 

With the exception of bowel symptoms, a significant postoperative reduction of all assessed symptoms was observed, irrespective of the mesh attachment point (cervix, uterus, vaginal apex). The rate of persistence was the highest for back pain (82.9%) and among the lowest for prolapse-related symptoms like feeling of downward pressure (27.7%) and foreign body sensation in the vagina (11%). Only 40% of the women in need for a pessary were still using it after surgery. The number of patients being affected by vaginal and bladder infections decreased from 31 (14.5%) and 33 (15.4%) patients to 14 (6.5%) and 19 (8.8%) patients postoperatively, respectively. After sacropexy, lower abdominal pain and pain severity were reduced significantly (*P* < 0.05). 

The number of patients suffering from fecal incontinence, constipation, and pain during defecation increased postoperative but not significantly, irrespective of the mesh attachment point. Whereas Bradley et al. [16] showed a statistically significant improvement of obstructive defecatory and other bowel symptoms after prolapse surgery and especially after sacropexy, Nygaard et al. [17] did not report improvement of bowel functions. Also, for the symptom constipation—before and after sacropexy—different results exist in the literature. Whereas Baessler and Schuessler [18] found significant improvement of constipation, Maher et al. [19] found no significant difference. This might indicate that other pathologic conditions besides rectocele influence bowel functions. This is also indicated by our finding that positive answers concerning defecation problems did not correlate with the extent of rectoceles in some cases, evaluated in postoperative followup. While 12 patients with postoperative rectocele report no defecation problems, 21 patients with no diagnosed rectocele suffer from defecation problems. However, these findings have to be interpreted carefully, as postoperative examination was 7.8 months and followup 24.5 months [20].

Furthermore, the correlation of the severity of bowel symptoms and of vaginal prolapse is discussed controversially. Weber et al. [21] found a weak correlation between bowel symptoms and severity of vaginal prolapse, whereas Meschia et al. [22] found a 2-fold increased risk of fecal incontinence in patients with a rectocele greater than grade II. In general, posterior procedures for rectocele repair in addition to sacropexy increase the risk of new bowel symptoms, including fecal incontinence with physical activity and pain prior to and during defecation [16, 23].

The number of patients with urinary incontinence, voiding difficulties, and feeling of residual urine decreased significantly postsurgically. But still about 60% of our patients showed persistent urinary symptoms. Higgs et al. [10] reported no change of urinary symptoms for half of their study population, and only 12% showed no urinary symptoms after surgery. Postoperative feeling of residual urine and voiding difficulties might be caused by a hypocontractile bladder due to sympathicus stimulation, as a result from surgical trauma [24]. These findings have important implications for counseling and treatment of patients regarding the prognosis of urinary and bowel symptoms after prolapse surgery.

The number of patients suffering from dyspareunia decreased significantly postoperatively with a persistence rate of 20%, supporting other studies [19, 25, 26]. Our study reports a higher success rate in resolved dyspareunia. Whereas preoperative dyspareunia was resolved in 79.6% of our patients; in the literature 58.4% [26] and 56% [19] were reported after abdominal sacropexy. This might be due to the operative technique, in which, on the one hand, the mesh is extraperitonealized, and on the other hand, due to the swab inserted into the vagina, the sutures do not push the net too far into the vaginal area. What is more, the vagina is fixated almost in good axial alignment, so it is only slightly moved laterally (preferably right-laterally, so as to gain distance to the Sigmoid colon).

In our study, we found a reduced sexual activity after surgery, correlating with age which is contrary to previous data which show an improved sexual activity irrespective of age [25]. While 47.1% of the patients indicated a reduced frequency of sex; 11.4% and 41.4% of the patients reported an increased frequency or no changes after surgery in our study.

In total, 24.7% of the patients suffered from recurrent symptoms 24.5 months after surgery, whereas an objective success rate of 96% according to the reoperation rate or 89.6% according to the persistence/recurrence prolapse assessment 7.9 months after surgery was reported for the same study population [20]. These data are comparable with the literature. Higgs et al. [10] reported recurrent/persistent urinary and bowel symptoms in 38% of patients 66 months after surgery. Rozet et al. [27] reported a symptomatic cure rate of 96% whereas 85% were anatomically cured.

Anatomical correction of the pelvic floor often does not correlate with subjective symptoms. Still nearly three-thirds of all patients were satisfied or very satisfied with the treatment outcome 2 years after surgery. 

There are a few limitations to our study. The percentage of followup loss was quite high with only 214 patients (69%) responding to the questionnaire. It is possible that mainly unsatisfied patients did not attend the followup which leads to falsified positive results. However, we assume the risk to be little as our data concerning symptoms and satisfaction are comparable to the literature. Our study is further limited by the fact that answers to the questionnaires were given in retrospect. It is assumed that some patients potentially embellish situations when asked to answer a question in retrospect, and, therefore, it is believed that retrospective studies attract and receive lower levels of participation. 

The third drawback of this study was the use of a non-validated symptoms questionnaire. Instead of using validated questionnaires, we tried to assess surgery outcome by symptoms according to specific definitions or criteria.

In future large population-based long-term follow-up studies, which focus on prolapse symptoms, using disease-specific validated questionnaires are necessary to draw a more definite conclusion about the subjective outcome of prolapse surgery.

## 5. Conclusion

Laparoscopic sacropexy is an efficient treatment for genital prolapse. We show a subjective success rate of 85.3% 24.5 months after surgery. With the exception of bowel symptoms, a significant postoperative reduction of all assessed symptoms was observed irrespective of the mesh attachment point. The high rate of persistent urinary and bowel symptoms requires special counseling and treatment for affected women.

##  Conflict of Interests

All authors have no coflict interests to declare.

## Figures and Tables

**Table 1:** Preoperative characteristics of the study population.

	Study group (*n* = 310)
Age (yrs, mean ± SD, range)	56.7 ± 10.2 (33–81)
Body mass index (kg/m^2^, mean + SD)	25.2 ± 3.51
Menopause (*n*, %)	159 (51.4)

ASA (*n*, %)	
ASA I	73 (23.5)
ASA II	135 (43.5)
ASA III	100 (32.3)
ASA IV	2 (0.6)

descensus (*n*, %)	
Stage I	73 (23.5)
Stage II	158 (51)
Stage III	76 (24.5)
Stage IV	3 (1)

Additional descensus (n, %)	
Anterior compartment	112 (35.1)
Posterior compartment	48 (15.5)

Urinary symptoms (*n*, %)	164 (52.9)
Stress incontinence	75 (45.7)
Urge incontinence	6 (3.6)
Mixed incontinence	10 (6.1)
No clear diagnosis	73 (44.5)

Other symptoms (*n*, %)	
Pressure on bladder	39 (12.6)
Pressure pain in vaginal area	133 (42.9)
Dyspareunia	23 (7.4)
Lower back pain	100 (32.3)
Pressure downwards directed	161 (51.9)

**Table 2:** Symptoms, preoperative and followup.

*n* = 214	Preoperative (*n*, %)	Followup (*n*, %)
Back pain	117 (54.6)	97 (45.3)
Pain in the lower abdomen	72 (33.6)	41 (19.5)
Defecation problems	59 (27.5)	69 (32.2)
Constipation	46 (21.5)	52 (24.3)
Pain during defecation	9 (4.2)	14 (6.5)
Fecal incontinence	7 (3.3)	10 (4.6)
Foreign body sensation in vagina	118 (55.1)	13 (11)
Prolapse-related symptoms	177 (82.7)	49 (22.8)
Dyspareunia	49 (24.3)	10 (5)
Urine incontinence	139 (65.3)	89 (41.5)
Feeling of residual urine	69 (32.2)	41 (19.1)
Voiding difficulties	43 (20.6)	24 (11.5)
Vaginal infections	31 (14.5)	14 (6.5)
Bladder infection	33 (15.4)	19 (8.8)
Need for pessary usage	20 (9.3)	8 (3.7)

**Table 3:** Lower abdominal pain, preoperative and followup.

*n* = 214	Preoperative (*n*, %)	Followup (%)
Pain, total	72 (100%)	41 (100%)
Mild	15 (20.8%)	17 (41.4%)
Medium	24 (33.3%)	16 (39%)
Severe	25 (34.7%)	8 (19.5%)
Very severe	8 (11.1%)	0

**Table 4:** Postoperative satisfaction.

*n* = 214	Patients (*n*, %)
Not satisfied	21 (9.8)
Partly satisfied	53 (24.7)
Satisfied	59 (27.6)
Very satisfied	81 (37.9)
